# Quantifying Sex Bias in Clinical Studies at Scale With Automated Data Extraction

**DOI:** 10.1001/jamanetworkopen.2019.6700

**Published:** 2019-07-03

**Authors:** Sergey Feldman, Waleed Ammar, Kyle Lo, Elly Trepman, Madeleine van Zuylen, Oren Etzioni

**Affiliations:** 1 Allen Institute for Artificial Intelligence, Seattle, Washington; 2 University of South Alabama College of Medicine, Mobile; 3 Department of Medical Microbiology and Infectious Diseases, University of Manitoba, Winnipeg, Manitoba, Canada

## Abstract

**Importance:**

Analyses of female representation in clinical studies have been limited in scope and scale.

**Objective:**

To perform a large-scale analysis of global enrollment sex bias in clinical studies.

**Design, Setting, and Participants:**

In this cross-sectional study, clinical studies from published articles from PubMed from 1966 to 2018 and records from Aggregate Analysis of ClinicalTrials.gov from 1999 to 2018 were identified. Global disease prevalence was determined for male and female patients in 11 disease categories from the Global Burden of Disease database: cardiovascular, diabetes, digestive, hepatitis (types A, B, C, and E), HIV/AIDS, kidney (chronic), mental, musculoskeletal, neoplasms, neurological, and respiratory (chronic). Machine reading algorithms were developed that extracted sex data from tables in articles and records on December 31, 2018, at an artificial intelligence research institute. Male and female participants in 43 135 articles (792 004 915 participants) and 13 165 records (12 977 103 participants) were included.

**Main Outcomes and Measures:**

Sex bias was defined as the difference between the fraction of female participants in study participants minus prevalence fraction of female participants for each disease category. A total of 1000 bootstrap estimates of sex bias were computed by resampling individual studies with replacement. Sex bias was reported as mean and 95% bootstrap confidence intervals from articles and records in each disease category over time (before or during 1993 to 2018), with studies or participants as the measurement unit.

**Results:**

There were 792 004 915 participants, including 390 470 834 female participants (49%), in articles and 12 977 103 participants, including 6 351 619 female participants (49%), in records. With studies as measurement unit, substantial female underrepresentation (sex bias ≤ −0.05) was observed in 7 of 11 disease categories, especially HIV/AIDS (mean for articles, −0.17 [95% CI, −0.18 to −0.16]), chronic kidney diseases (mean, −0.17 [95% CI, −0.17 to −0.16]), and cardiovascular diseases (mean, −0.14 [95% CI, −0.14 to −0.13]). Sex bias in articles for all categories combined was unchanged over time with studies as measurement unit (range, −0.15 [95% CI, −0.16 to −0.13] to −0.10 [95% CI, −0.14 to −0.06]), but improved from before or during 1993 (mean, −0.11 [95% CI, −0.16 to −0.05]) to 2014 to 2018 (mean, −0.05 [95% CI, −0.09 to −0.02]) with participants as the measurement unit. Larger study size was associated with greater female representation.

**Conclusions and Relevance:**

Automated extraction of the number of participants in clinical reports provides an effective alternative to manual analysis of demographic bias. Despite legal and policy initiatives to increase female representation, sex bias against female participants in clinical studies persists. Studies with more participants have greater female representation. Differences between sex bias estimates with studies vs participants as measurement unit, and between articles vs records, suggest that sex bias with both measures and data sources should be reported.

## Key Points

**Question:**

What is the magnitude of female underrepresentation in clinical studies?

**Findings:**

In this cross-sectional study, machine reading to extract sex data from 43 135 published articles and 13 165 clinical trial records showed substantial underrepresentation of female participants, with studies as measurement unit, in 7 of 11 disease categories, especially HIV/AIDS, chronic kidney diseases, and cardiovascular diseases. Sex bias in articles for all categories combined was unchanged over time with studies as the measurement unit but improved with participants as measurement unit.

**Meaning:**

This study suggests that sex bias against female participants in clinical studies persists, but results differ when studies vs participants are the measurement units.

## Teaser

This cross-sectional study uses machine reading to estimate the magnitude of female underrepresentation in clinical studies worldwide.

## Introduction

For proper application of clinical study results, enrolled participants should represent the populations for which treatments are intended. When female patients receive treatment based on the results of studies of male participants, unanticipated adverse events may occur because of sex-specific differences in disease patterns, metabolism, and drug pharmacokinetics and clearance.^1,2^ Health risks were greater in female patients than in male patients for 8 of 10 prescription drugs withdrawn from the US market from 1997 to 2000.^3^ The slower metabolism of the insomnia drug zolpidem in female patients than in male patients may have contributed to multiple zolpidem-related motor vehicle crashes before the recommended dose was decreased in female patients by 50%.^4,5,6^ Female patients may experience more adverse drug reactions, more disease and disability, later diagnosis, less aggressive treatment, and lower case survival rates for some diseases than male patients.^7,8,9^

The National Institutes of Health Revitalization Act of 1993 established legal requirements and guidelines to ensure the inclusion of female participants and racial/ethnic minority participants in clinical research.^10^ However, underrepresentation of female participants in studies relative to disease prevalence (known as *enrollment sex bias* or *sex bias*) persists.^11,12^ In treatment trials of 11 non–sex-specific cancers (9671 patients), underrepresentation of female participants was noted in trials of 3 cancer types.^13^ In 120 randomized clinical trials (total, 160 801 participants) in 12 specialties, 24.6% of participants were female, with no improvement observed in sex-balanced enrollment or sex-specific analyses.^11^ From 2000 to 2002, female participants had lower enrollment fraction—defined as the number of trial participants divided by the estimated number of cancer cases in the population—than male participants for colorectal (total, 8434 participants) and lung cancer (4297 participants) trials.^12^ A literature search for 1999 to 2018 showed 13 major analyses of sex bias in clinical studies, but these analyses were limited in size (range, 36-865 studies and 2339-398 801 participants) and disease categories and were performed with manual methods or analysis of isolated data sets (eAppendix and eTable 1 in the Supplement).^12,13,14,15,16,17,18,19,20,21,22,23,24^

Computerized, automated data extraction (also known as *machine reading*) of published research articles enables the development of large, complex systems to organize, integrate, and communicate information from numerous studies.^25,26,27,28,29^ However, a literature search did not show previous studies of machine automation for quantifying sex bias in clinical studies at the national or global scale.

The purpose of this study was to develop a scalable automated machine reading method to extract sex data from numerous clinical studies and analyze sex bias in published articles and clinical trial records at scale.^30,31^ We hypothesized that computerized data extraction from numerous articles and records may provide comprehensive and longitudinal information about sex bias in clinical studies at scale.

## Methods

### Data Sources

We analyzed the number of male and female participants in clinical studies that were identified and extracted in electronic searches from 2 sources on December 31, 2018: (1) published articles from the search engine Semantic Scholar, which had 41 million articles indexed, including more than 20 million full-text articles and all articles in PubMed Central from 1966 to 2018,^30,32^ and (2) clinical trial records in the Aggregate Analysis of ClinicalTrials.gov (AACT) database, which contained metadata for 288 515 studies registered at ClinicalTrials.gov in 205 countries from 1999 to 2018.^33,34^

Global disease prevalence data for male and female participants were obtained from the Global Health Data Exchange (GHDx), a database synthesized from multiple data sources, including scientific literature and population representative surveys.^35,36^ Prevalence values for selected disease categories defined by GHDx were obtained from an online catalog of health-related data (eTable 2 in the Supplement).^35^

This study was not considered human subjects research according to the Federal Policy for the Protection of Human Subjects because it was a secondary analysis of data from published articles and trial records. Therefore, the study was not submitted for institutional review board approval.

### Study Sample and Data Extraction

We identified all articles related to clinical studies in PubMed using article categories selected from the XML PubMed publication type attribute <PublicationTypeList> (1 038 324 articles) (eTable 3 in the Supplement).^37^ Semantic Scholar accessed the full text of 388 227 articles (37%). We restricted the analysis to articles about medical disorders by including only articles labeled with any Medical Subject Headings (MeSH) terms under “disease,” “vaccination,” “disorder,” “pathological,” or “neoplasms” in the MeSH taxonomy tree, and processed these articles with optical character recognition (OmniPage; Nuance Communications) (295 139 articles). As the analysis was based on automated extraction of male and female participant numbers from tables, we included articles with at least 1 table extracted (249 845 articles).

We developed an algorithm (PubMed-Extract) to extract articles and sex data from tables of articles in portable document format (eTable 4 in the Supplement). PubMed-Extract was designed to parse the tables, identify relevant semantics of rows and columns by matching patterns, and aggregate information across table rows and columns (eAppendix in the Supplement). We limited the analysis to 11 GHDx disease categories for which morbidity frequency data were available in GHDx and more than 1000 articles were identified: cardiovascular diseases, diabetes, digestive diseases, hepatitis (types A, B, C, and E), HIV/AIDS, kidney diseases (chronic), mental disorders, musculoskeletal disorders, neoplasms, neurological disorders, and respiratory diseases (chronic). We mapped articles to disease categories using the MeSH terms associated with each article (eTable 5 in the Supplement). In the 249 845 articles that were processed by optical character recognition and had at least 1 table extracted, 147 807 articles (59%) were mapped to at least 1 disease category, from which PubMed-Extract extracted male and female participant numbers in 43 135 articles (17%).

We developed another algorithm (AACT-Query) to extract sex data from tables in AACT records that could be queried with Structured Query Language. We identified AACT records of 33 361 studies that had male and female participant numbers. After excluding incomplete studies, there were 28 187 studies. After mapping records to disease categories using MeSH terms, we retained 13 165 records (47%) that mapped to at least 1 disease category, and used AACT-Query to extract male and female participant numbers.

### Variables

Female prevalence fraction (F-Prev) for each disease category was defined as the fraction of female participants in the disease category and was estimated by dividing the global morbidity count for female participants by global morbidity count for both male and female participants using GHDx data. Female participant fraction (F-Particip) was defined as the fraction of female participants among all participants who were included in the studies, and was estimated 2 ways: with (1) studies as measurement units, by computing the ratio of female participants to all participants for each study and determining the simple average of this ratio for all studies without any weighting by study size and (2) participants as measurement units, by dividing the total number of female participants in all studies by the total number of male and female participants in all studies combined. The female participant fraction was estimated from articles using PubMed-Extract and records using AACT-Query. The primary outcome variable was enrollment sex bias in clinical studies, defined as F-Particip minus F-Prev (values for sex bias ranged from −1 to 1, with 0 indicating no bias; negative sex bias indicates that female participants were represented less than male participants).

### Accuracy of PubMed-Extract Estimates

We evaluated the accuracy of sex bias estimates from PubMed-Extract by comparing them with the true F-Particip that was determined from manually extracted numbers of male and female participants from 100 randomly selected articles. Mean absolute error was calculated by averaging the absolute difference between the PubMed-Extract estimates and true value of F-Particip in individual articles.

We evaluated the recall of PubMed-Extract, defined as the percentage of articles for which PubMed-Extract produced the exact number of male and female participants as manually extracted in another random set of 100 articles on cardiovascular diseases. Mean absolute error was sensitive to severity of estimation errors, whereas recall equally penalized all estimation errors.

### Comparison Between PubMed-Extract and AACT-Query

To evaluate differences between sex bias estimated with PubMed-Extract vs AACT-Query, we analyzed studies that were represented in both estimates. We identified 1400 articles for which (1) PubMed-Extract produced numerical estimates of sex bias, (2) the articles were linked each to exactly 1 AACT record, (3) the AACT record included numbers of male and female participants, and (4) the full text of the articles was available through PubMed. We compared the numbers of male and female participants between these articles and records and manually inspected a sample of 50 discordant articles and records to determine the reasons for discrepancies. We contacted study authors for comments when we were unable to determine reasons for discrepancies.

### Statistical Analysis

For each disease category, we computed 1000 bootstrap estimates of sex bias by resampling individual studies with replacement. Sex bias was reported as mean and 95% bootstrap confidence interval, determined from the bottom 2.5% and top 97.5% of bootstrap estimate percentiles. The *P* value for the null hypothesis of zero sex bias was equal to the probability of type I error corresponding to the widest confidence interval that contained zero. We calculated *P* values under the null hypothesis by repeating the bootstrap confidence interval procedure over a fine grid of confidence levels (decreasing from 99.999%), taking the smallest confidence level whose interval contained zero; the *P* value was the probability of type I error = 2 × (1 − confidence level). For each disease category and time period, statistical significance for a hypothesis test for sex bias was defined by *P* ≤ .001 using 2-tailed tests.

For analysis of sex bias in articles vs time, we fitted an intercept-only linear model to sex bias values before or during 1993 and subsequent 5-year increments separately with studies and participants as measurement unit and plotted estimated intercept coefficients vs time with error bars representing 95% confidence intervals for the mean coefficient. We assumed Gaussian distribution because bootstrapping was precluded by dividing the data into 5-year increments.

The association between estimated sex bias and number of participants in each study was evaluated with fixed-effects linear regression, with number of participants defined as a categorical variable with 10 equal-sized bins (eTable 6 in the Supplement). We controlled for publication year (continuous variable) and disease category (categorical variable). Analyses were performed with the statistical functions of the Python programming language, version 3.6 (Python Software Foundation).

## Results

There were 792 004 915 participants, including 390 470 834 female participants (49%), in articles and 12 977 103 participants, including 6 351 619 female participants (49%) in records. The F-Prev was highest for digestive diseases and lowest for hepatitis (Table). With studies as measurement unit, substantial female underrepresentation (sex bias ≤ −0.05) in articles and records was observed in 7 of 11 disease categories, including HIV/AIDS (mean for articles, −0.17 [95% CI, −0.18 to −0.16]), kidney diseases (chronic) (mean, −0.17 [95% CI, −0.17 to −0.16]), cardiovascular diseases (mean, −0.14 [95% CI, −0.14 to −0.13]), neoplasms, digestive diseases, neurological disorders, and hepatitis (Table). The only category with female overrepresentation was musculoskeletal disorders (Table).

**Table.** Sex Bias in Clinical Studies Determined From Published Articles and Clinical Trial Records^a^

Disease Category	Global Female Prevalence Fraction	Measurement Unit	Published Articles			AACT Records		
			Studies or Participants, No.	Female Participant Fraction	Sex Bias (95% CI)	Studies or Participants, No.	Female Participant Fraction	Sex Bias (95% CI)
Cardiovascular	0.51	Studies	14 371	0.37	−0.14 (−0.14 to −0.13)^b^	2164	0.41	−0.10 (−0.11 to −0.09)^b^
		Participants	540 050 700	0.49	−0.02 (−0.06 to −0.01)	2 229 071	0.39	−0.12 (−0.15 to −0.08)^b^
Diabetes	0.48	Studies	3727	0.45	−0.03 (−0.03 to −0.02)^b^	1420	0.46	−0.03 (−0.03 to −0.02)^b^
		Participants	38 420 434	0.48	0.00 (−0.05 to 0.04)	4 823 058	0.47	−0.01 (−0.08 to 0.02)
Digestive	0.60	Studies	1282	0.49	−0.11 (−0.12 to −0.10)^b^	348	0.54	−0.06 (−0.08 to −0.04)^b^
		Participants	8 519 928	0.51	−0.09 (−0.13 to −0.07)^b^	147 821	0.56	−0.03 (−0.06 to −0.01)
Hepatitis A, B, C, and E	0.44	Studies	1131	0.34	−0.09 (−0.10 to −0.09)^b^	632	0.37	−0.06 (−0.07 to −0.05)^b^
		Participants	1 833 724	0.37	−0.06 (−0.17 to 0.06)	243 846	0.39	−0.05 (−0.07 to −0.03)^b^
HIV/AIDS	0.50	Studies	1741	0.33	−0.17 (−0.18 to −0.16)^b^	387	0.27	−0.23 (−0.25 to −0.21)^b^
		Participants	30 459 386	0.53	0.02 (−0.09 to 0.06)	155 531	0.35	−0.15 (−0.20 to −0.11)^b^
Kidney, chronic	0.57	Studies	2554	0.40	−0.17 (−0.17 to −0.16)^b^	476	0.42	−0.15 (−0.16 to −0.13)^b^
		Participants	18 747 970	0.44	−0.13 (−0.18 to −0.09)^b^	201 763	0.42	−0.15 (−0.17 to −0.12)^b^
Mental	0.48	Studies	3635	0.47	−0.01 (−0.02 to 0.00)^b^	1650	0.44	−0.04 (−0.05 to −0.03)^b^
		Participants	58 097 584	0.48	−0.01 (−0.19 to 0.07)	463 645	0.49	0.00 (−0.01 to 0.02)
Musculoskeletal	0.56	Studies	2418	0.66	0.10 (0.09 to 0.11)^b^	983	0.70	0.14 (0.13 to 0.15)^b^
		Participants	5 898 338	0.60	0.03 (0.00 to 0.08)	438 112	0.65	0.09 (−0.05 to 0.18)
Neoplasms	0.51	Studies	11 121	0.40	−0.11 (−0.11 to −0.11)^b^	3179	0.41	−0.10 (−0.11 to −0.10)^b^
		Participants	54 377 430	0.49	−0.03 (−0.04 to −0.01)^b^	2 946 236	0.50	−0.02 (−0.09 to 0.03)
Neurological	0.59	Studies	3431	0.50	−0.09 (−0.10 to −0.09)^b^	1338	0.52	−0.07 (−0.08 to −0.06)^b^
		Participants	10 576 242	0.53	−0.06 (−0.09 to −0.03)^b^	497 964	0.65	0.06 (−0.01 to 0.12)
Respiratory, chronic	0.48	Studies	2800	0.43	−0.04 (−0.05 to −0.04)^b^	1161	0.44	−0.03 (−0.04 to −0.02)^b^
		Participants	116 410 829	0.48	0.00 (−0.05 to 0.02)	1 231 162	0.47	−0.01 (−0.04 to 0.01)
Total^c^	0.54	Studies	48 211	0.42	−0.12 (−0.12 to −0.11)^b^	13 738	0.45	−0.09 (−0.09 to −0.08)^b^
		Participants	883 392 565	0.49	−0.05 (−0.06 to −0.03)^b^	13 378 210	0.48	−0.06 (−0.09 to −0.03)^b^

Abbreviation: AACT, Aggregate Analysis of ClinicalTrials.gov.

^a^ Data as of December 31, 2018. Published articles from 1966 to 2018 in PubMed were obtained using a search engine (Semantic Scholar)^30,32^; clinical trial records from 1999 to 2018 were obtained from the AACT database.^33^ Global prevalence data were obtained from the Global Health Data Exchange.^35^ Sex bias with studies as measurement unit was defined as female participant fraction with studies as units (mean ratio of female participants/[male participants + female participants] for each study) minus female prevalence fraction, and is shown in rows with number of studies; sex bias with participants as measurement unit was defined as female participant fraction with participants as units (ratio of total number of female participants in all studies/total number of participants in all studies combined) minus female prevalence fraction, and is shown in rows with number of participants. Sex bias range was −1 to 1, with 0 indicating no bias; negative sex bias indicates that female participants were represented less than male participants. Sex bias (1000 bootstrap estimates) is reported as mean and 95% bootstrap confidence interval (bottom 2.5%, top 97.5%).

^b^ Difference between sex bias value vs 0: *P* ≤ .001.

^c^ Totals include duplicate use of studies that mapped to more than 1 disease category. There were 38 506 of the 43 135 published articles (89%), representing 706 161 955 of the 792 004 915 participants (89%), and 12 609 of the 13 165 AACT records (96%), representing 12 636 768 of the 12 977 103 participants (97%), that mapped to a single disease category; only 4629 published articles (11%), representing 85 842 960 participants (11%), and 556 AACT records (4%), representing 340 335 participants (3%), contributed to sex bias estimates for more than 1 disease category.

With participants as measurement unit, sex bias against female participants in articles was highest for chronic kidney diseases and lowest for musculoskeletal disorders and HIV/AIDS, and in records was highest for HIV/AIDS, chronic kidney diseases, and cardiovascular diseases. Sex bias usually was less negative when the measurement unit was participants vs studies (eg, for articles about cardiovascular disease with participants as the measurement unit, mean sex bias was −0.02 [95% CI, −0.06 to −0.01]; with studies as the measurement unit, mean sex bias was −0.14 [95% CI, −0.14 to −0.13]) (Table). Most articles and records mapped to a single disease category (Table).

**Figure 1.**
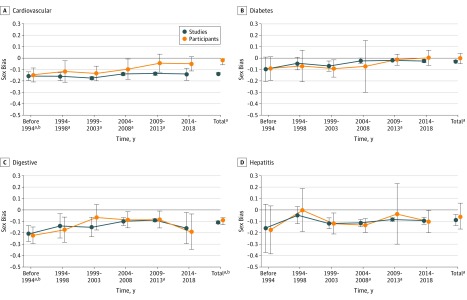
Sex Bias in Clinical Studies Over Time Determined From Published Articles for Cardiovascular Diseases, Diabetes, Digestive Diseases, and Hepatitis (Types A, B, C, and E) An intercept-only linear model was fitted to sex bias values from before and during 1993 and subsequently in 5-year increments. Estimated sex bias intercept coefficients were plotted against time for studies (blue) and participants as measurement unit (orange), with error bars representing 95% confidence intervals for the mean coefficients. The points for total at the right of each graph represent the mean sex bias totals for each category. Sex bias was defined as female participant fraction (determined separately for studies and participants as measurement unit) minus female prevalence fraction (values for sex bias ranged from −1 to 1, with 0 indicating no bias; negative sex bias indicates that female participants were represented less than male participants). ^a^Difference between sex bias value vs 0; *P* < .001 for studies as measurement unit. ^b^Difference between sex bias value vs 0; *P* < .001 for participants as measurement unit.

**Figure 2.**
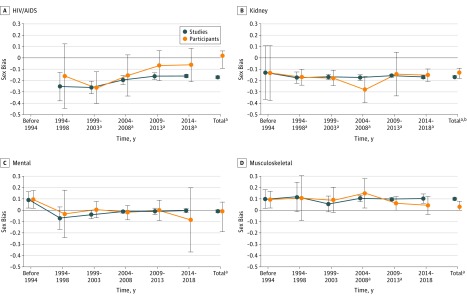
Sex Bias in Clinical Studies Over Time Determined From Published Articles for HIV/AIDS, Kidney Diseases (Chronic), Mental Disorders, and Musculoskeletal Disorders An intercept-only linear model was fitted to sex bias values from before and during 1993 and subsequently in 5-year increments. Estimated sex bias intercept coefficients were plotted against time for studies (blue) and participants as measurement unit (orange), with error bars representing 95% confidence intervals for the mean coefficients. For HIV/AIDS before or during 1993, sex bias values for studies (−0.40) and participants (−0.42) were not plotted because they were based on only 3 articles (total, 138 participants). Sex bias was defined as female participant fraction (determined separately for studies and participants as measurement unit) minus female prevalence fraction (values for sex bias ranged from −1 to 1, with 0 indicating no bias; negative sex bias indicates that female participants were represented less than male participants). ^a^Difference between sex bias value vs 0; *P* < .001 for studies as measurement unit. ^b^Difference between sex bias value vs 0; *P* < .001 for participants as measurement unit.

**Figure 3.**
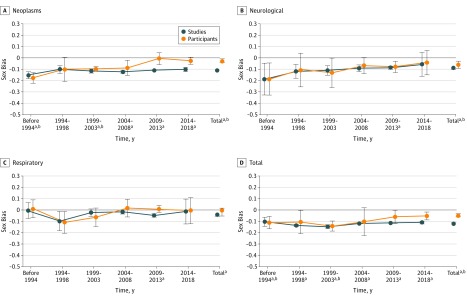
Sex Bias in Clinical Studies Over Time Determined From Published Articles for Neoplasms, Neurological Disorders, Respiratory Diseases (Chronic), and Total (All Categories Combined) An intercept-only linear model was fitted to sex bias values from before and during 1993 and subsequently in 5-year increments. Estimated sex bias intercept coefficients were plotted against time for studies (blue) and participants as measurement unit (orange), with error bars representing 95% confidence intervals for the mean coefficients. The total number of published articles (all categories combined) increased from before or during 1993 (total, 482 articles) to 2014 to 2018 (18 627 articles). Sex bias in articles for all categories combined was unchanged over time with studies as measurement unit (range, −0.15 [−0.16 to −0.13] to −0.10 [−0.14 to −0.06]), but improved from before 1993 (−0.11 [−0.16 to −0.05]) to 2014 to 2018 (−0.05 [−0.09 to −0.02]) with participants as measurement unit. Sex bias was defined as female participant fraction (determined separately for studies and participants as measurement unit) minus female prevalence fraction (values for sex bias ranged from −1 to 1, with 0 indicating no bias; negative sex bias indicates that female participants were represented less than male participants). ^a^Difference between sex bias value vs 0; *P* < .001 for studies as measurement unit. ^b^Difference between sex bias value vs 0; *P* < .001 for participants as measurement unit.

With studies as measurement unit, sex bias was stable from before or during 1993 to 2018 for most disease categories (Figure 1, Figure 2, and Figure 3). With participants as measurement unit, sex bias improved (became less negative by ≥0.10) over time for cardiovascular diseases, HIV/AIDS, neoplasms, and neurological disorders (Figure 1, Figure 2, and Figure 3). Sex bias in articles for all categories combined was unchanged over time with studies as measurement unit (range, −0.15 [95% CI, −0.16 to −0.13] to −0.10 [95% CI, −0.14 to −0.06]), but improved from before 1993 (mean, −0.11 [95% CI, −0.16 to −0.05]) to 2014 to 2018 (mean −0.05 [95% CI, −0.09 to −0.02]) with participants as the measurement unit.

The mean absolute error between true F-Particip from data extracted manually vs automatically (PubMed-Extract) was 0.008. Errors made by PubMed-Extract were caused when (1) the table varied from typical table organization, (2) there were 2 or more columns for total counts and no single column for grand total, and (3) there were optical character recognition errors such as incorrect merging of multiple columns or splitting of single columns (eTable 4 in the Supplement). Manual analysis of automatically extracted participant numbers showed that 14 of 100 articles evaluated did not report the number of male and female participants, PubMed-Extract returned correct numerical estimates for 43 of the other 86 articles (recall, 50%), and mean precision for exact row extraction of male and female numbers was 0.75.

Comparison of the 1400 studies that had both articles and records showed that 675 studies (48%) had numbers of male and female participants that differed between articles and records, with magnitude of the difference between studies ranging from a minimum of 35 participants (52% of participants in the AACT record) to a maximum of 15 746 participants (92%). In 50 studies selected randomly from the 675 discordant studies, manual evaluation showed that discrepancies between articles and records were caused because the article was based on a subset of the trial data in the record (19 studies), PubMed-Extract extractions were incorrect or from the wrong table (14 studies), the article reported the number of participants who completed the trial vs the record that included enrolled participants who did not complete the trial (7 studies), the article was published before completion of the trial (3 studies), there was author error (1 study), and the article included patients from multiple trials (1 study); in 5 studies, the causes of discrepancies were unknown despite contacting authors for comments. In 6 of the 50 studies, the reasons for discrepancies were provided through email communication with study authors.

Linear regression with fixed effects to evaluate the association between publication year, disease category, and study size and sex bias in articles showed that the coefficients for number-of-participants deciles were positive and different from zero for the fifth decile (121-188 participants) through 10th decile (≥2990 participants), indicating that larger study size was associated with greater female representation (eTable 6 in the Supplement).

## Discussion

Using a large amount of data from articles and records, we observed substantial female underrepresentation in studies for diverse disease categories, especially HIV/AIDS and chronic kidney diseases. There was little increase in female representation in studies from before or during 1993 to 2018 using studies as measurement unit but improved female representation with participants as measurement unit (Figure 1, Figure 2, and Figure 3). Most disease categories were not evaluated previously (eTable 1 in the Supplement). The algorithms provided an effective and accurate automated scalable method for extracting male and female participant numbers and enabled expansion of analyses about sex bias to varied disease categories and integration of new data.

**Figure 4.**
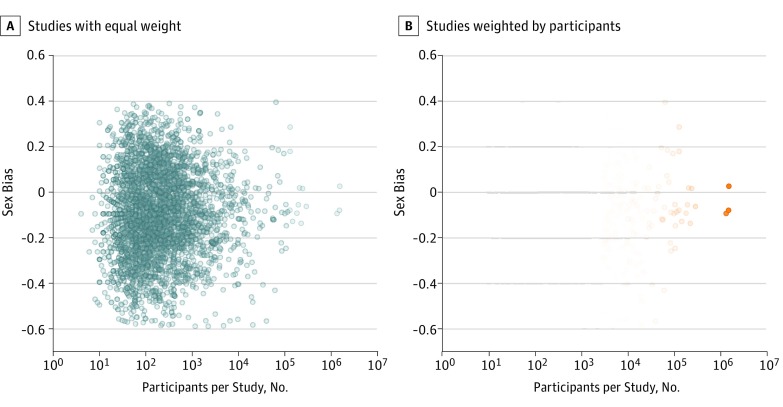
Sex Bias vs Number of Study Participants for 14 371 Cardiovascular Clinical Studies, Estimated From Published Articles by the PubMed-Extract Algorithm Each point represents 1 article. A, With studies as the measurement unit of sex bias, each study point has equal intensity of blue shade and contribution to the overall estimate of sex bias. B, With participants as the measurement unit of sex bias, study point orange shade intensity is proportional to the number of participants; small studies are essentially invisible and contribute little to the overall sex bias estimate.

Previous studies of sex bias used studies or participants, but not both, as measurement unit (eTable 1 in the Supplement). With studies as measurement unit, each study has an equal contribution to the overall sex bias estimate, regardless of study size, providing a study-by-study evaluation of sex bias (Table, Figure 4). In contrast, with participants as measurement unit, participants may have an equal contribution to the overall sex bias estimate, providing a population estimate; however, larger studies contribute proportionally more, and smaller studies have a nearly invisible contribution to overall sex bias estimates (Figure 4). The marked difference in sex bias in articles with studies vs participants as measurement unit for cardiovascular diseases (−0.14 vs −0.02) and neoplasms (−0.11 vs −0.03) is evidence that sex bias determined with both measurement units should be reported, and that sex bias results may be less sensitive to female underrepresentation with participants than studies as measurement unit (Table, Figure 1, Figure 2, and Figure 3). The use of studies as measurement unit may ensure that small studies of less prevalent diseases receive equal representation in estimates of overall sex bias (Figure 4). The limited change in sex bias over time for all categories combined with studies as measurement unit (Figure 3) may be addressed with policy and funding initiatives that focus on sex bias regardless of proposed study size. Furthermore, the importance of study size was underscored by the relation between study size and female representation in articles (eTable 6 in the Supplement).

With studies as measurement unit, sex bias estimates from articles and records were consistent in polarity and magnitude for diabetes, HIV/AIDS, kidney diseases, mental disorders, neoplasms, neurological disorders, and respiratory diseases but differed in magnitude for digestive diseases and musculoskeletal disorders (Table). Differences in sex bias estimates may, in part, be due to having fewer records than articles (digestive diseases, 348 records vs 1282 articles), and AACT data may have been biased geographically because trial registration requirements for ClinicalTrials.gov may apply only to US clinical trials.^34^ Geographic differences may be important because of marked variation in regional disease profiles, such as differences in HIV/AIDS incidence between sub-Saharan Africa vs East Asia.^38^ Future studies may include machine reading algorithms to evaluate study location.

Differences in sex bias estimates between articles vs records also may be due to discrepancies in male and female participant numbers between articles and records observed in 48% of studies. Manual evaluation of these discrepancies was limited to 50 studies because it was time-consuming and associated with delays inherent with email queries to authors when reasons for discrepancies could not be ascertained from the article and record. A previous comparison of randomized clinical drug trials in ClinicalTrials.gov vs counterpart published articles concluded that trial results should be evaluated systematically from both sources because of important differences, including more complete reporting in records than articles, variation in reporting between articles from specialty vs general journals, and absence of an article corresponding to 50% of trials posted on ClinicalTrials.gov (so-called abandoned trials).^39,40^ Trial registration and reporting on ClinicalTrials.gov may vary between studies funded by industry or government sources, and the requirement of mandatory posting of trial results on ClinicalTrials.gov within 1 year of completion of data collection is adhered to infrequently and may promote the posting of cursory reports that may include inaccurate or incomplete data that are not peer reviewed.^6,41,42,43^ Journal publication may be associated with partial and altered reporting (so-called filtered data) due to space limitations, publication bias, revised analyses and data exclusion due to suggestions from peer reviewers, and delays inherent in journal submission and peer review.^40,41^ The observation of sex bias differences between articles and records is further evidence to support the need for greater transparency and accuracy in trial reporting in both media.

The comparison of data from articles vs records may have been affected by our decision to include data from articles about studies other than clinical trials, such as observational studies, case series studies, and quality improvement analyses. Although a focus on trials alone may provide a more direct comparison between data from articles vs records, the inclusion of all published articles may provide a more realistic description of current sex bias in funded and nonfunded clinical research. Observational studies may be considered lower in evidence quality than trials but remain important because they provide valuable context for trial results and data in areas with limited trials.^44,45,46^ Furthermore, randomized trials may not necessarily represent general disease populations because of participant exclusion criteria.^47^ Nevertheless, sex bias estimates for trials alone may be determined in future work by applying different filters to the data extraction algorithms.

In selecting disease categories that previously were defined in GHDx, we recognized potential overlap between categories, such as cardiovascular, kidney, or neurological diseases in studies of patients who had diabetes. Nevertheless, the disease categories were used because they represented large, important, clinically relevant categories. Most studies were limited to only 1 of the 11 disease categories, and only 11% of articles and 4% of records contributed to sex bias estimates for more than 1 disease category (Table). The attribution of cost and resource allocation to overlapping disease categories is an inherent issue in epidemiology and public health that we addressed by specifying the sources of disease category definitions and data and quantifying the number of studies that mapped to more than 1 category.^48^

### Limitations

Limitations of the present study include the analysis of sex bias without other variables. Sex bias may vary with age for colorectal and lung cancer^12^; further evaluation using our algorithms may enable robust analysis of the interaction between sex, age, and race in study enrollment. We did not evaluate diagnoses that have marked variation of sex prevalence within disease categories, such as different types of cancer (eg, breast vs prostate cancer), because our goal was to provide a broad overview about sex bias for different disease categories; in future work, filters added to the data extraction code may enable more focused sex bias data for specific diseases. In addition, we included participant counts from primary studies and secondary analyses such as meta-analyses and systematic reviews, but in estimating sex bias, we did not account for multiple inclusion of the same primary study participants in the secondary analyses; therefore, estimates of sex bias from articles may have been affected preferentially by primary studies that were included in secondary analyses, and the magnitude of this effect is unknown. The total number of more than 792 million participants may seem unrealistically high because it may imply that 10% of the 7.7 billion people globally were involved in a clinical study; the large number of participants may have been affected by large population-based studies including a survey from China (381 million participants) and study of death records from the United States, England, and Wales (almost 86 million participants) that accounted for 467 million participants (53%).^49,50^ In future big data studies that are based on articles, it may be advisable to modify the data extraction coding to exclude duplicate use of studies and analyze large outlier studies separately. For the time series, we used publication date of articles and did not extract information about the time range of study execution; that may be considered in future work.

## Conclusions

Automated extraction of participant numbers in clinical reports provides an effective alternative to manual analysis of demographic bias and may expedite analyses for multiple diseases globally. Our findings indicate that studies with more participants have greater female representation. However, sex bias against female participants in clinical studies persists despite legal and policy initiatives to increase female representation.

## Notes

Supplement. **eAppendix.** Quantifying Sex Bias in Clinical Studies at Scale With Automated Data Extraction **eReferences** **eTable 1.** Previous Studies About Enrollment Sex Bias in Clinical Research **eTable 2.** Global Disease Category Prevalence **eTable 3.** Categories in the XML PubMed Field <PublicationTypeList> Used to Identify Study-Related Articles in PubMed **eTable 4.** Example Table Used for Illustration of Sex Data Extraction Using PubMed-Extract **eTable 5.** Relation Between Disease Category and Medical Subject Headings Terms **eTable 6.** Linear Model for Number of Participants vs Enrollment Sex Bias, Controlling for Publication Year and Disease Category
