# Supplementary material for: Quantifying Sex Bias in Clinical Studies at Scale With Automated Data Extraction
Source: JAMA Netw Open. 2019 Jul 3;2(7):e196700. doi: 10.1001/jamanetworkopen.2019.6700 (PMC6613296; doi:10.1001/jamanetworkopen.2019.6700)
Supplement: Supplement. — eAppendix. Quantifying Sex Bias in Clinical Studies at Scale With Automated Data Extraction eReferences eTable 1. Previous Studies About Enrollment Sex Bias in Clinical Research eTable 2. Global Disease Category Prevalence eTable 3. Categories in the XML PubMed Field Used to Identify Study-Related Articles in PubMed eTable 4. Example Table Used for Illustration of Sex Data Extraction Using PubMed-Extract eTable 5. Relation Between Disease Category and Medical Subject Headings Terms eTable 6. Linear Model for Number of Participants vs Enrollment Sex Bias, Controlling for Publication Year and Disease Category [file jamanetwopen-2-e196700-s001.pdf]

# Supplementary Online Content

Feldman S, Ammar W, Lo K, Trepman E, van Zuylen M, Etzioni O. Quantifying sex bias in clinical studies at scale with automated data extraction. *JAMA Netw Open*. 2019;2(7):e196700. doi:10.1001/jamanetworkopen.2019.6700

**eAppendix.** Quantifying Sex Bias in Clinical Studies at Scale With Automated Data Extraction
**eReferences**
**eTable 1.** Previous Studies About Enrollment Sex Bias in Clinical Research
**eTable 2.** Global Disease Category Prevalence
**eTable 3.** Categories in the XML PubMed Field <PublicationTypeList> Used to Identify Study-Related Articles in PubMed
**eTable 4.** Example Table Used for Illustration of Sex Data Extraction Using PubMed-Extract
**eTable 5.** Relation Between Disease Category and Medical Subject Headings Terms
**eTable 6.** Linear Model for Number of Participants vs Enrollment Sex Bias, Controlling for Publication Year and Disease Category

This supplementary material has been provided by the authors to give readers additional information about their work.

## eAppendix: Quantifying Sex Bias in Clinical Studies at Scale With Automated Data Extraction

### Previous Studies of Sex Bias in Clinical Research

After the National Institutes of Health (NIH) Revitalization Act of 1993 was signed into United States law, several studies quantified the degree to which women were included in clinical research, with varied results (eTable 1).[1-13] Most of the previous studies about sex bias in cancer trials were limited in scope to trials conducted at a single facility or for 1 specialty or had other constraints. In cancer trials conducted by the Southwest Oncology Group, overall female participant enrollment was proportional to prevalence, but analysis for cancer type showed a significant difference between enrollment and prevalence for women in 3 of 11 non–sex-specific cancer types studied – colorectal cancer, head and neck cancer, and lymphoma.[1] A follow-up study of therapeutic nonsurgical colorectal and lung cancer trials between 2000 and 2002 showed that women were underrepresented for both cancer types.[5] When breast cancer trials were included in the analysis, women were overrepresented.[7,11]

Analyses of other single-specialty trials included cardiovascular trials funded by the National Heart, Lung, and Blood Institute, and showed that women were underrepresented in mixed-sex trials.[3] Although women were overrepresented in migraine treatment trials,[12] women were underrepresented in vascular surgery randomized controlled trials, especially smaller, non-government-funded and single-center trials.[8] Two analyses of randomized controlled trials in multiple specialties that were started after 1993 and received public funding also showed that women were underrepresented, especially in drug trials, but these analyses included only 46 and 56 clinical trials.[6,9]

In addition to enrollment sex bias, sex bias exists in outcomes analysis; more than two-thirds of NIH-funded studies with women participants that were published in major medical journals from 1993 to 1998 did not analyze the data by sex, and there was no improvement over the period studied.[4] Other factors that may affect enrollment in clinical trials include the increased enrollment in cancer trials in patients who have fee-for-service insurance coverage,[2] and lower enrollment in patients who had lower income or age ≥ 65 years.[1,14]

### Disease Category Prevalence

Disease categories with highest global prevalence included neurological, musculoskeletal, and mental disorders (eTable 2).

### Code for Obtaining Disease Counts

Code was written and applied to obtain data from the Aggregate Analysis of ClinicalTrials.gov (AACT) for computation of sex bias estimates (https://github.com/allenai/pubmedextract).[15] Numbers obtained from application of this AACT-Query code may vary with time because the AACT database is updated regularly. An AACT account is required to access data, including proper signing in from the user and password fields.

### Parsing Tables for Sex

Articles related to clinical research were identified from PubMed using article categories selected from the XML PubMed publication type attribute <PublicationTypeList> (1 038 324 articles) (eTable 3).[16] The input to PubMed-Extract was a set of tables from full published articles parsed by an optical character recognition application (OmniPage, Nuance Communications). The output from PubMed-Extract was (1) estimated numbers of female and male participants or (2) an error message. The output was accompanied by diagnostic messages.

The requirements of the PubMed-Extract procedure were illustrated with an example table to show key regularities observed that made the procedure tractable (eTable 4). It was necessary to have table information organized in rows, with some participant row headers defined by terms indicating sex such as male, female, men, or women. PubMed-Extract required arithmetic consistency within tables; in the example, the sum of male and female participants had to equal the total number of participants. In some instances, tables were excluded because of arithmetic inconsistency caused by numeric rounding; e.g., 3069 participants × 44% = 1350 participants, not 1355 participants. PubMed-Extract allowed participant numbers to be spread across multiple columns such as columns representing different treatments (e.g., Treatment A, Treatment B, Placebo, Total; no. of participants for Treatment A + Treatment B + Placebo = Total no. of participants). Furthermore, PubMed-Extract enabled various formats for table cells that contained information about numbers of participants, e.g., 20, 20%, 20.2, 20.2%, 20/30, 20 (50.2%), 20 (50), 20/30, or (66%).

**PubMed-Extract Functions: Extraction of the Number of Male and Female Participants from Tables**

PubMed-Extract extracted female and male counts for each table that had been parsed within an article using 3 functions.

(1) subdivide: To extract participant sex counts, we needed to know which rows and columns contained or did not contain sex counts. We specified which rows and columns had headers that contained text information that indicated the type of row or column. This function subdivided each table into 3 sections: a row header section involving 0 or more of the first columns, a column header section involving 0 or more of the first rows, and the numerical portion of the table below and to the right of the row and column headers (eTable 4). There typically was an additional section at the top left corner of the table, at the intersection of the row and column headers, that we discarded because it was uninformative (eTable 4).

(2) parse_sex_rows: This function evaluated each cell in the table, performed a series of regular expression transformations (known as *regex transformations*), and extracted number and percentage of participants. Rows that had any successfully parsed values were retained for further analysis. In the example table, the 2 rows that had headings for *P* values would be deleted (eTable 4).

(3) extract_male_female_counts_from_table: This function combined information from individual cells to create a single set of counts per table, by finding which rows contained female and male counts, extracting male and female counts for each column within those rows, and combining these counts across columns.

Subsequently, PubMed-Extract selected the number of participants from tables that had extracted numbers for male and female participants and the most convincing regularity. The function extract_male_female_counts_from_table provided a notification that summarized the column and row regularity condition, such as the following outputs: (1) all columns added up to another column, and there was a single column named *total* column (e.g., there were columns such as *Treatment A*, *Placebo*, and *Total*, and the counts for *Treatment A* and *Placebo* added up to the *Total*); (2) all columns added up to another column, and there was not a single column named *total* column (e.g., there were columns such as *Treatment A*, *Placebo*, and *Otherwise Named Column*, and the counts for *Treatment A* and *Placebo* added up to the *Otherwise Named Column* that was not obviously a *Total* column by name, but it happened to be the exact or near sum of the other 2 columns); (3) all columns added up to a number of participants smaller than the biggest *total* column; or (4) there were multiple named *total* columns and all but 1 of these columns added up to a column of grand totals. The table that had the most highly ranked diagnostic message was selected as the table from which the final participant numbers were used. Ties in ranking of tables were resolved by choosing the table that appeared earliest in the article, because Table 1 often contained the number of participants.

**Distant Supervision with Aggregate Analysis of ClinicalTrials.gov**

The table parsing core of the PubMed-Extract algorithm was not our first choice for table parsing. A limitation of PubMed-Extract is that we did not have a source of ground truth. The original idea was to connect published articles with their AACT records, and use these AACT records as ground truth. This would have enabled us to (1) train a machine learning algorithm (that needs ground truth) and (2) evaluate

the accuracy of PubMed-Extract automatically instead of relying on manual, time-consuming annotations. Therefore, we initially attempted to extract participant numbers from text instead of tables of articles using distant supervision from the AACT records, to enable the use of information from the AACT records as a guide for extracting counts from published articles – a single method of data extraction from published articles and AACT records – but this was unsuccessful.

As an artificial example of this method, the AACT database might have reported that there were 41 women and 52 men participants in a hypothetical clinical trial. Although we considered the method of searching for all articles in PubMed that linked to this AACT trial identification number and had full article text from Semantic Scholar, and searching for the numbers 41 and 52 in the article text, this method was unsuccessful because (1) numbers such as 41 and 52 appeared in more places than expected, (2) the numbers of participants frequently were in tables and not text, and (3) the parse of the published article file was noisy, precluding clean extraction. The numbers of participants in the AACT records did not match those reported in the published article for 48% of a subset of 1400 studies (Results).

**Use of Medical Subject Heading Terms to Map Articles to Disease Categories**

The selection of the quantity of 250 Medical Subject Heading (MeSH) terms was arbitrary and based on the perceived tradeoff between fewer numbers of MeSH terms that might not be sufficient for mapping vs too many MeSH terms that might require too much processing time. For the 250 MeSH terms, 167 MeSH terms (67%) mapped to the 11 disease categories, and 83 MeSH terms (33%) did not map to any disease category (eTable 5). With 250 MeSH terms, 147 807 articles were mapped to ≥ 1 disease category, resulting in 43 135 articles that enabled extraction of the numbers of male and female participants, judged to be sufficient for this study. The use of more MeSH terms would have yielded more articles, at the expense of greater processing time.

## eReferences


1. Hutchins LF, Unger JM, Crowley JJ, Coltman CA Jr, Albain KS. Underrepresentation of patients 65 years of age or older in cancer-treatment trials. *N Engl J Med*. 1999;341(27):2061-2067. doi: 10.1056/NEJM199912303412706
2. Klabunde CN, Springer BC, Butler B, White MS, Atkins J. Factors influencing enrollment in clinical trials for cancer treatment. *South Med J*. 1999;92(12):1189-1193. doi:10.1097/00007611- 199912000-00011
3. Harris DJ, Douglas PS. Enrollment of women in cardiovascular clinical trials funded by the National Heart, Lung, and Blood Institute. *N Engl J Med*. 2000;343(7):475-480. doi: 10.1056/NEJM200008173430706
4. Vidaver RM, Lafleur B, Tong C, Bradshaw R, Marts SA. Women subjects in NIH-funded clinical research literature: lack of progress in both representation and analysis by sex. *J Womens Health Gend Based Med*. 2000;9(5):495-504. doi: 10.1089/15246090050073576
5. Murthy VH, Krumholz HM, Gross CP. Participation in cancer clinical trials: race-, sex-, and age-based disparities. *JAMA*. 2004;291(22):2720-2726. doi: 10.1001/jama.291.22.2720
6. Geller SE, Adams MG, Carnes M. Adherence to federal guidelines for reporting of sex and race/ethnicity in clinical trials. *J Womens Health (Larchmt)*. 2006;15(10):1123-1131. doi: 10.1089/jwh.2006.15.1123
7. Stewart JH, Bertoni AG, Staten JL, Levine EA, Gross CP. Participation in surgical oncology clinical trials: gender-, race/ethnicity-, and age-based disparities. *Ann Surg Oncol*. 2007;14(12):3328-3334. doi: 10.1245/s10434-007-9500-y
8. Hoel AW, Kayssi A, Brahmanandam S, Belkin M, Conte MS, Nguyen LL. Under-representation of women and ethnic minorities in vascular surgery randomized controlled trials. *J Vasc Surg*. 2009;50(2):349-354. doi: 10.1016/j.jvs.2009.01.012
9. Geller SE, Koch A, Pellettieri B, Carnes M. Inclusion, analysis, and reporting of sex and race/ethnicity in clinical trials: have we made progress? *J Womens Health (Larchmt)*. 2011;20(3):315-320. doi: 10.1089/jwh.2010.2469.
10. Polit DF, Beck CT. Is there still gender bias in nursing research? An update. *Res Nurs Health*. 2013;36(1):75-83. doi: 10.1002/nur.21514
11. Ibrahim M, Ogunleye F, Roye J, Yadav S, Townsel D, Yu Z. Representation of minorities and elderly in cancer clinical trials at a single institution – the William Beaumont Hospital experience. *J Cancer Epidemiol Prev*. 2017;2(1):1.
12. Robbins NM, Bernat JL. Minority representation in migraine treatment trials. *Headache*. 2017;57(3):525-533. doi: 10.1111/head.13018
13. Kalliainen LK, Wisecarver I, Cummings A, Stone J. Sex bias in hand surgery research. *J Hand Surg Am*. 2018;43(11):1026-1029. doi: 10.1016/j.jhsa.2018.03.026
14. Unger JM, Hershman DL, Albain KS, Moinpour CM, Petersen JA, Burg K, Crowley JJ. Patient income level and cancer clinical trial participation. *J Clin Oncol*. 2013;31(5):536-542. doi: 10.1200/JCO.2012.45.4553
15. Aggregate Analysis of ClinicalTrials.gov (AACT) database. Clinical Trials Transformation Initiative (CTTI) Web site. https://www.ctti-clinicaltrials.org/aact-database. Accessed November 11, 2018.
16. United States National Library of Medicine. PubMed. 24. <PublicationTypeList>. In: MEDLINE®PubMed® XML Element Descriptions and their Attributes. National Institutes of Health Web site. https://www.nlm.nih.gov/bsd/licensee/elements_descriptions.html#publicationtypelist. Accessed November 29, 2018.

## eTables

## eTable 1. Previous Studies About Enrollment Sex Bias in Clinical Research*

| Measurement Unit | Study | Disease Category | Study Size | Data Source |
|---|---|---|---|---|
| Participants | | | **No. of Participants** | |
| | Hutchins (1999) | Cancer | 16 396 | SWOG |
| | Klabunde (1999) | Cancer | 2339 | NCI Clinical Trial database |
| | Harris (2000) | Cardiovascular | 398 801 | NHLBI database |
| | Murthy (2004) | Cancer | 75 215 | NCI Clinical Trial database |
| | Stewart (2007) | Cancer | 13 991 | NCI Clinical Trial database |
| | Ibrahim (2017) | Cancer | 55 408 | 1 facility |
| Studies | | | **No. of Studies** | |
| | Vidaver (2000) | Various | 865 | Cochrane, NIS database |
| | Geller (2006) | General Internal Medicine | 46 | Manual |
| | Hoel (2009) | Vascular | 52 | Manual |
| | Geller (2011) | General Internal Medicine | 56 | Manual |
| | Polit (2013) | Nursing | 300 | Manual |
| | Robbins (2017) | Migraine | 36 | Manual |
| | Kalliainen (2018) | Hand Surgery | 335 | Manual |

*Studies were identified as part of the literature review. ***Abbreviations***: Manual, manual review of published articles; NCI, National Cancer Institute; NHLBI, National Heart, Lung, and Blood Institute; NIS, National Inpatient Sample; SWOG, Southwest Oncology Group.

## eTable 2. Global Disease Category Prevalence*

| Disease Category | Male Prevalence | Female Prevalence |
|---|---:|---:|
| Cardiovascular | 230 151 198.37 | 240 660 147.73 |
| Diabetes mellitus | 198 739 363.51 | 184 713 651.61 |
| Digestive | 105 392 477.75 | 155 531 129.82 |
| Hepatitis A, B, C, and E | 361 556 015.33 | 278 670 135.19 |
| HIV/AIDS | 18 006 418.92 | 18 206 619.16 |
| Kidney, chronic | 119 062 132.67 | 156 867 666.57 |
| Mental | 572 376 387.79 | 537 698 169.26 |
| Musculoskeletal | 554 703 882.15 | 711 567 522.98 |
| Neoplasms | 20 472 968.92 | 21 518 329.72 |
| Neurological | 1 050 076 288.94 | 1 542 054 073.48 |
| Respiratory, chronic | 299 323 606.02 | 272 396 741.15 |

*Prevalence was reported as the estimated number of patients in the world. Global prevalence data were obtained from the Global Health Data Exchange (2016 edition). The Global Health Data Exchange used model-based estimates, so the outputs were decimals and not whole numbers.

**eTable 3. Categories in the XML PubMed Field <PublicationTypeList> Used to Identify Study-Related Articles in PubMed***

| <PublicationTypeList> Category |
|---|
| Adaptive Clinical Trial |
| Clinical Study |
| Clinical Trial |
| Clinical Trial, Phase I |
| Clinical Trial, Phase II |
| Clinical Trial, Phase III |
| Clinical Trial, Phase IV |
| Collected Works |
| Controlled Clinical Trial |
| Equivalence Trial |
| Multicenter Study |
| Observational Study |
| Pragmatic Clinical Trial |
| Randomized Controlled Trial |
| Study Characteristics |
| Twin Study |
| Validation Studies |

*XML PubMed field no. 24 <PublicationTypeList>.[16]

## eTable 4. Example Table Used for Illustration of Sex Data Extraction Using PubMed-Extract*

| **Variable** | | **Total (N = 3069)** | | **Self-report Quit** | **Covalidated Quit** |
|---|---|---|---|---|---|
| | | **No. of Participants (%)** | | **%** | **%** |
| Age (y) | | | | | |
| | 16-24 | 330 | (11) | 41 | 25 |
| | 25-34 | 676 | (22) | 53 | 38 |
| | 35-44 | 760 | (25) | 55 | 42 |
| | 45-54 | 631 | (21) | 59 | 47 |
| | 55-64 | 458 | (15) | 65 | 57 |
| | 65-85 | 214 | (7) | 69 | 61 |
| | *P* ≤ | | | .001 | .001 |
| Sex | | | | | |
| | Male | 1355 | (44) | 59 | 45 |
| | Female | 1714 | (56) | 55 | 53 |
| | *P* ≤† | | | NS | NS |

*N = 3069 participants. Shading: green, row headers; blue, column headers; yellow, numerical portion. The top left corner of the table, at the intersection of the row and column headers, was discarded because it was uninformative.

†NS, not significant (*P* > .001).

## eTable 5. Relation Between Disease Category and Medical Subject Headings Terms*

| Disease Category | No. of MeSH Terms | 3 Most Frequent MeSH Terms in the Disease Category |
|---|---|---|
| Cardiovascular | 36 | Hypertension; stroke; myocardial infarction |
| Diabetes mellitus | 10 | Diabetes mellitus, type 2; diabetes mellitus, type 1; insulin resistance |
| Digestive | 8 | Gastroesophageal reflux; Crohn disease; gastrointestinal diseases |
| Hepatitis A, B, C, and E | 4 | Hepatitis C, chronic; hepatitis C; hepatitis B |
| HIV/AIDS | 3 | HIV infections; acquired immunodeficiency syndrome; HIV seropositivity |
| Kidney, chronic | 7 | Kidney failure, chronic; renal insufficiency, chronic; kidney diseases |
| Mental | 15 | Schizophrenia; mental disorders; depressive disorder |
| Musculoskeletal | 13 | Arthritis, rheumatoid; osteoarthritis, knee; low back pain |
| Neoplasms[†] | 44 | Breast neoplasms; neoplasms; lung neoplasms |
| Neurological | 18 | Cognition disorders; Parkinson disease; Alzheimer disease |
| Respiratory, chronic | 9 | Asthma; pulmonary disease, chronic obstructive; pneumonia |
| None of the above | 83 | Postoperative complications; disease-free survival; disease progression |
| Total | 250 | Postoperative complications; breast neoplasms; disease-free survival |

*N = 250 Medical Subject Headings (MeSH) terms.
[†]Although some neoplasms occur primarily in 1 sex, we did not include single-sex trials in the analyses.

## eTable 6. Linear Model for Number of Participants vs Enrollment Sex Bias, Controlling for Publication Year and Disease Category*

| Variable Name | Category Name or No. of Participants | Coefficient | (95% Confidence Interval) | *P* |
|---|---|---|---|---|
| Intercept | | –0.2047 | (–0.218 to –0.191) | $4.6 \times 10^{-187}$ |
| Disease category | Cardiovascular | Reference category | | |
| | Diabetes mellitus | 0.1104 | (0.104 to 0.116) | $2.8 \times 10^{-285}$ |
| | Digestive | 0.032 | (0.022 to 0.042) | $4.5 \times 10^{-11}$ |
| | Hepatitis A, B, C, and E | 0.0429 | (0.033 to 0.053) | $7.7 \times 10^{-17}$ |
| | HIV/AIDS | –0.0353 | (–0.044 to –0.027) | $6.0 \times 10^{-17}$ |
| | Kidney, chronic | –0.0281 | (–0.035 to –0.021) | $3.7 \times 10^{-15}$ |
| | Mental | 0.1286 | (0.123 to 0.135) | 0 |
| | Musculoskeletal | 0.2375 | (0.23 to 0.245) | 0 |
| | Neoplasms | 0.0308 | (0.027 to 0.035) | $1.1 \times 10^{-46}$ |
| | Neurological | 0.0455 | (0.039 to 0.052) | $1.8 \times 10^{-46}$ |
| | Respiratory, chronic | 0.0953 | (0.089 to 0.102) | $4.7 \times 10^{-169}$ |
| No. of participants | 2 to 32 | Reference category | | |
| | 33 to 53 | 0.0044 | (–0.002 to 0.011) | .191[†] |
| | 54 to 81 | 0.0077 | (0.001 to 0.014) | .022[†] |
| | 82 to 120 | 0.0085 | (0.002 to 0.015) | .013[†] |
| | 121 to 188 | 0.0129 | (0.006 to 0.02) | .0001 |
| | 189 to 299 | 0.0225 | (0.016 to 0.029) | $4.2 \times 10^{-11}$ |
| | 300 to 505 | 0.0265 | (0.02 to 0.033) | $8.4 \times 10^{-15}$ |
| | 506 to 1003 | 0.0288 | (0.022 to 0.035) | $3.4 \times 10^{-17}$ |
| | 1004 to 2989 | 0.0183 | (0.012 to 0.025) | $8.8 \times 10^{-8}$ |
| | ≥ 2990 | 0.0317 | (0.025 to 0.039) | $3.9 \times 10^{-20}$ |
| Year of publication | | 0.0575 | (0.042 to 0.073) | $1.1 \times 10^{-13}$ |

*Reported as coefficient (95% confidence interval).
[†]Not significant (*P* > .001).
